# Atmospheric Black Carbon Loadings and Sources over Eastern Sub-Saharan Africa Are Governed by the Regional Savanna Fires

**DOI:** 10.1021/acs.est.2c05837

**Published:** 2022-10-30

**Authors:** Leonard Kirago, Örjan Gustafsson, Samuel M. Gaita, Sophie L. Haslett, H. Langley deWitt, Jimmy Gasore, Katherine E. Potter, Ronald G. Prinn, Maheswar Rupakheti, Jean de Dieu Ndikubwimana, Bonfils Safari, August Andersson

**Affiliations:** † Department of Environmental Science, Stockholm University, 10691Stockholm, Sweden; ‡ Bolin Centre for Climate Research, Stockholm University, 10691Stockholm, Sweden; § Center for Global Change Science, Massachusetts Institute of Technology, 54-1312, Cambridge, Massachusetts02139, United States; ∥ Climate Secretariat, Ministry of Education, 622Kigali, Rwanda; ⊥ Physics Department, School of Physics, College of Science and Technology, University of Rwanda, 4285Kigali, Rwanda; # Institute for Advanced Sustainability Studies (IASS), 14467Potsdam, Germany

**Keywords:** source apportionment, carbon isotopes, Savanna fires, relative emission factors

## Abstract

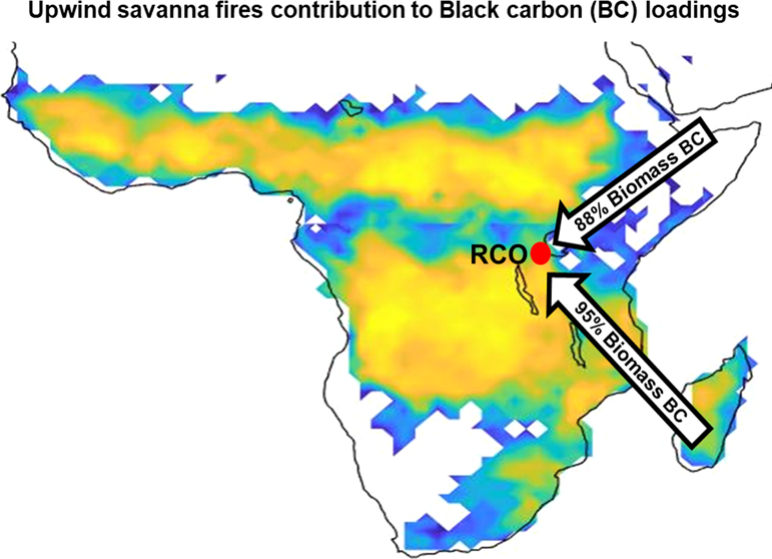

Vast black carbon (BC) emissions from sub-Saharan Africa are perceived to warm the regional climate, impact rainfall patterns, and impair human respiratory health. However, the magnitudes of these perturbations are ill-constrained, largely due to limited ground-based observations and uncertainties in emissions from different sources. This paper reports multiyear concentrations of BC and other key PM_2.5_ aerosol constituents from the Rwanda Climate Observatory, serving as a regional receptor site. We find a strong seasonal cycle for all investigated chemical species, where the maxima coincide with large-scale upwind savanna fires. BC concentrations show notable interannual variability, with no clear long-term trend. The Δ^14^C and δ^13^C signatures of BC unambiguously show highly elevated biomass burning contributions, up to 93 ± 3%, with a clear and strong savanna burning imprint. We further observe a near-equal contribution from C3 and C4 plants, irrespective of air mass source region or season. In addition, the study provides improved relative emission factors of key aerosol components, organic carbon (OC), K^+^, and NO_3_^–^, in savanna-fires-influenced background atmosphere. Altogether, we report quantitative source constraints on Eastern Africa BC emissions, with implications for parameterization of satellite fire and bottom-up emission inventories as well as regional climate and chemical transport modeling.

## Abstract 2

In this paper, we show that the concentrations of health- and climate-affecting black carbon air particles in the sub-Saharan African atmosphere are dominated by emissions from savanna fires.

## Introduction

Sub-Saharan Africa (SSA) is a global hotspot for aerosol emissions.^1−3^ A large but poorly constrained contribution is from large-scale regional fires, mainly lit by humans and occasionally triggered by lightning strikes.^2^ The region accounts for 70% of the global burned area and over 50% of the global carbonaceous aerosol budget.^4−7^ Aerosols generally have a short atmospheric residence time (days-week); hence, there is large spatio-temporal variability in aerosol concentrations. Consequently, the associated impact on climate, human health, and biogeochemical cycles is primarily regional.^8−11^ Despite the fact that the SSA region is one of the world’s largest sources of aerosol emissions, the aerosol characteristics and sources in the SSA region are poorly constrained in comparison with other geographical regions, largely owing to limited ground-based observations.^1,12^

Black carbon (BC) is a strong light-absorbing aerosol and thereby contributes to climate warming.^13,14^ However, the climate effects of BC are associated with several large uncertainties, including relative emission strengths, atmospheric distribution and transport, and optical properties.^15,16^ For example, the BC emissions are typically estimated using bottom-up emission inventories, which serve as a primary input in many modeling studies. These emission estimates are typically highly uncertain (a factor of 2–3), and even more so in SSA due to poorly constrained activity and emission factors (EFs).^2,6,17,18^ Alternative approaches such as satellite-derived emission data have been shown to improve model skill, but are in turn challenged by uncertain parametrizations for aerosol species such as BC, and by limited geographical coverage.^5,19,20^ Overall, detailed and regional-scale estimates of emissions and observational data, including aerosol concentrations, properties, and source-specific emission tracers, are needed to improve regional models and to combat the vast regional emissions.

A high-precision understanding of BC source contribution may be achieved using carbon isotope signatures. The carbon-14 signature of BC (often reported as Δ^14^C) allows differentiation between biomass burning and fossil fuel combustion with high precision and specificity. The Δ^14^C may be combined with the stable carbon signature (δ^13^C) to further resolve biomass and/or fossil sources into more detailed categories.^20−22^ Isotope characterization of BC allows refinement of model studies and emission inventories, while also providing complementary and unambiguous source information from observational data.^19,20^ So far, the number of applications of carbon isotope techniques in Africa has been limited to one study of BC in urban Nairobi and to bulk carbonaceous aerosols (i.e., unresolved total carbon) at the Rwanda Climate Observatory.^1,18^ Therefore, isotope-based source constraints for BC at a background location in SSA—currently unavailable—would substantially improve the understanding of BC aerosol emissions on a regional scale.

In this study, we present a multiyear (2014–2019) study of BC and the chemical composition of PM_2.5_ aerosols at the Rwanda Climate Observatory, a strategically located regional background site located on top of Mt. Mugogo. The dual-carbon isotope signatures (δ^13^C and Δ^14^C) of BC were investigated for a full year to understand the BC source profile and to contribute to reducing the large uncertainties in regional BC emission inventories.

## Materials and Methods

### Aerosol Sample Collection at Rwanda Climate Observatory

The Rwanda Climate Observatory (RCO) is a mountaintop monitoring site for greenhouse gases and aerosols (1.586° S, 29.566° E; 2590m a.s.l.), and is a site in the network of the Advanced Global Atmospheric Gases Experiment (AGAGE; https://agage.mit.edu).^23^ Site characteristics and meteorology are detailed elsewhere.^1,24^ A high-volume sampler (flowrate at 30 m^3^ h^–1^; model DH-77, DIGITEL Elektronik AG, Switzerland) installed at the station, 5 m above ground level, was used to collect PM_2.5_ aerosols on pre-combusted quartz fiber filters (400 °C for 5 h to remove organic matter; 15 cm in diameter). In this study, night-time-only (01.00–06.00 h) filter samples were collected over a 7-day period, between May 2014 and April 2016. The high-altitude location of RCO captures a free tropospheric environment and regional background atmospheric conditions during night-time when the station is less influenced by the planetary boundary layer aerosol regime.^1,24^ The collected filter samples and monthly field blanks were shipped to Stockholm University and analyzed for water-soluble inorganic species, carbonaceous aerosols, and carbon isotopes of the BC fraction.

### Chemical and Isotopic Analyses

Water-soluble inorganic ions were extracted using 18 M-ohm Milli-Q water by ultrasonication, and concentrations were determined by Dionex Aquion ion chromatography (IC; Thermo Scientific). Extracted cations (e.g., K^+^, Na^+^, NH_4_^+^, Ca^2+^, and Mg^2+^) were separated using Dionex IonPac CS12A separation column and 20 mM methane sulfonic acid eluent, while for anions, Dionex IonPac AS22-Fast separation column was used with a 4.2 mM Na_2_CO_3_ and 1.7 mM NaHCO_3_ mixture as the eluent. The IC instrument was calibrated using commercial standards (Merck KGaA), and several samples were analyzed in triplicate. The field blank contributions to K^+^, NH_4_^+^, SO_4_^2+^, and NO_3_^–^ were at a maximum of 7%, while Ca^2+^, Mg^2+^, and Cl^–^ concentrations were lower and occasionally close to detection limits (Supporting Information, SI Table S1).

A thermal-optical transmission carbon analyzer (Sunset Laboratory, Tigard, OR) was used to measure the carbonaceous aerosols—organic carbon (OC) and BC (measured as its mass-based analogue, often called elemental carbon, EC)—using the NIOSH 5040 protocol described in detail elsewhere.^25,26^ Briefly, the more volatile OC is combusted in the stepwise temperature protocol and in an inert (He) environment, while the recalcitrant BC is evolved under an oxidizing (He–O_2_ mixture) environment. The carbon analyzer detector response was calibrated using a sucrose standard, and the instrument’s long-term performance was monitored using in-house standards, traceable to the NIST-8785 urban dust Standard Reference Material. The OC values were blank-corrected, while the BC content in field blanks was below detection limits. Triplicate analyses were used to evaluate measurement precision and sample deposition homogeneity and were found to be within 5% of the mean concentration value.

The BC fraction of the carbonaceous aerosols was isolated, cryo-trapped, and analyzed for dual-carbon isotopes, following a previously described methodology.^21,27^ Twenty samples collected during high BC loading events in the dry season (June–August and December–February) were used (SI Figure S1). The BC loadings during the wet seasons were insufficient for isotope analysis of BC. Prior to BC isolation, sample punches used were acid-fumigated to eliminate carbonates, and the OC-BC split time was determined for each sample. The filter punches were then combusted in the carbon analyzer, and the CO_2_ evolved from the BC fraction was diverted to a cryogenic trap, purified to remove moisture (using anhydrous Mg(ClO_4_)_2_) and halogen-/sulfur-containing gases (by heated silver wool at 500 °C), and collected in glass ampules.^18,27^ The samples were sent to the Tandem Laboratory at the Department of Nuclear Physics at Uppsala University for isotopic characterization. The δ^13^C signatures were measured using an isotope ratio mass spectrometer, while radiocarbon signatures were measured using accelerator mass spectrometry.^28,29^

During the NIOSH 4050 protocol, a fraction of OC is commonly pyrolyzed during the helium phase, inadvertently creating “black carbon”. As OC often has a different isotopic signature compared to BC, this pyrolyzed carbon (PryC) pool may perturb the estimated isotopic signature of BC.^18,27,30^ To resolve such an impact on the present dataset, we employ a sensitivity analysis, where we assume the observed Δ^14^C is contaminated by a fraction of PyrC. Using a mass balance criterion, we can then estimate the Δ^14^C for “real” BC. Based on our previous study for RCO, the Δ^14^C for PyrC (TC) may be estimated as +37‰.^1^ Assuming that the typical Δ^14^C for BC at RCO is −32‰, we then find that if as much as 30% of the BC is of PyrC origins, the Δ^14^C of real BC is −61‰. This would correspond to a shift in the estimated fraction biomass by 3% (see next section and SI Table S2), indicating that PyrC does not impact our conclusions significantly.

### Source Apportionment Calculations

The Δ^14^C signature may be used to compute the fraction biomass (*f*_bio_), using the assumption of isotopic mass balance, eq 1.
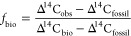
1Here, Δ^14^C_obs_ is the observed value, Δ^14^C_bio_ is the value of the biomass source endmember, and Δ^14^C_fossil_ is the fossil endmember. As fossil materials are entirely depleted in ^14^C, the Δ^14^C_fossil_ endmember is −1000‰. The Δ^14^C_bio_ is more complicated, as it (via photosynthesis) reflects the Δ^14^C signature of atmospheric CO_2_, which varies significantly over time. Nuclear bomb tests in the 1960s strongly elevated the ^14^C signature of CO_2_, which is on a steady decline due to the combustion of fossil fuels (Suess effect).^31^ For Africa, the Δ^14^C_bio_ has been estimated to be +57 ± 52‰ for the current study period.^1^

By combining Δ^14^C with the stable carbon isotope signature (δ^13^C), it is possible to resolve the sources of BC into detailed categories.^20,32,33^ For Africa, the main sources of carbonaceous aerosols are C_3_ plants (e.g., woody plants), C_4_ plants (e.g., some savanna grasses), and liquid fossil combustion (e.g., gasoline and oil).^1,7^ The fractional source contributions (*f*_C3_, *f*_C4_, and *f*_fossil_, respectively) may then be determined according to the following isotopic mass balance
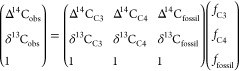
2Here, Δ^14^C_C3_ is +57 ± 52‰, while Δ^14^C_C4_ is +20 ± 10‰ as most C_4_ plants are annual, and therefore reflect the ^14^C signature of CO_2_ at the time of collection (2014/15). The δ^13^C endmember signatures for this region are estimated as: δ^13^C_C3_ = −27.1 ± 2.0‰; δ^13^C_C4_ = −16.6 ± 2.2‰; and δ^13^C_fossil_ = −25.3 ± 1.3‰.^1^ To account for the natural variability in the endmember, we use a Bayesian source apportionment framework.^1^ The source fractions and their uncertainties were estimated through Markov chain Monte Carlo (MCMC) simulations, implemented in Matlab 2019b (100.0000 iterations; 10.000 burn-in; 100 data thinning).

### Analysis of Aethalometer Data

High-temporal-resolution equivalent BC data (eBC; optical-based BC commonly referred to as eBC)^34^ was retrieved from an AE33 Aethalometer at 880 nm (Magee Scientific, Inc.) and binned into hourly resolution. Spikes in the eBC data, potentially from short-term pollution events, were removed following the sliding window algorithm (SI note S1). The resulting eBC data was compared to the Sunset Laboratory thermo-optical BC measurements (SI Note S1). Overall, the aethalometer measurements were found to be higher by a factor of 3.2 relative to thermo-optical BC measurements, possibly due to absorption enhancement of the aged plumes intercepted at RCO.^35^ De-trended eBC data (dividing hourly data, 01.00–06.00 h, by the 7 days weekly floating average) showed a mono-modal log-normal concentration distribution (SI Figure S3). This is expected from single exponential dynamics (e.g., sink), suggesting that the night-time dynamics on a time scale shorter than a week represent fluctuations during atmospheric transport.^36^

### Satellite Observations and Air Mass Back Trajectories

The regional and seasonal fires were captured with remote sensing fire-spot data retrieved from the NASA Fire Information for Resource Management Services (FIRMS) database.^37^ Backward air mass trajectory (BTs) analysis to determine the air mass transport pathways and potential source regions was carried out using the HYSPLIT (version 4) model.^38,39^ Hourly, 5-day BTs were computed for an arrival height at RCO of 100 m, including wet deposition along the trajectory.

## Results and Discussion

### Aerosol Characteristics at RCO

**Figure 1:**
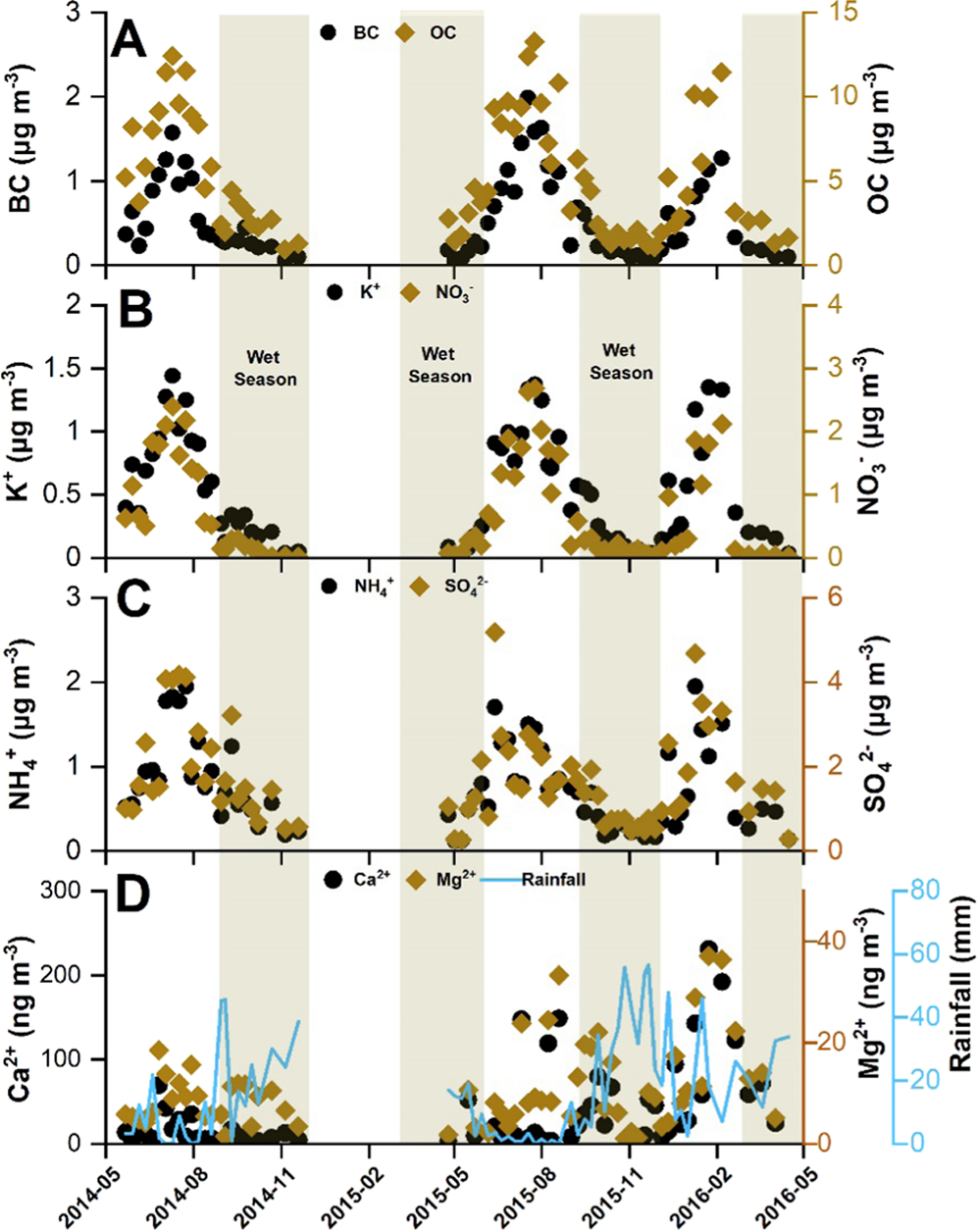
Temporal variations in mass concentrations of the main constituents of PM_2.5_ aerosols at Rwanda Climate Observatory during 2014–2016 period. The investigated PM_2.5_ species exhibit strong seasonality, with low aerosol concentrations observed during the wet seasons (highlighted with gray background). Data gap exists between December 2014 and April 2015 due to instrument failure after a lightning strike.

All PM_2.5_ aerosol species investigated here—carbonaceous aerosols and water-soluble inorganic species—exhibit a strong seasonality (Figure 1). Overall, low aerosol concentrations are observed during the wet periods (March–May and September–November), while peak concentrations occur during the intersecting dry periods. The relative contribution of carbonaceous aerosols (CA = 2.2*OC + BC) to the total aerosol mass is consistently high, at around 70% throughout the year (SI Figure S4). The highest fractional variability was noted for NO_3_^–^, which increased from ∼2% of PM_2.5_ mass during the wet season to 6% during the dry period, while mean seasonal concentrations increased 7-fold (SI Table S3). NO_3_^–^, typically associated with lightning strikes and traffic emissions, has been found elevated during savanna burning episodes, suggesting that large amounts are likely produced due to savanna fires.^1,4,40,41^ Crustal elements (Mg^2+^ and Ca^2+^), and Cl^–^, were mostly close to/below detection limits, except during events associated with air masses originating from Saharan dust plumes. Cl^–^ has been found to be elevated in fresh savanna smoke but not in long-range transported plumes.^40,42^ While Cl^–^ also originates from sea salt, the sea salt contribution is low (<2%) at this inland site (SI Note S2).

**Figure 2:**
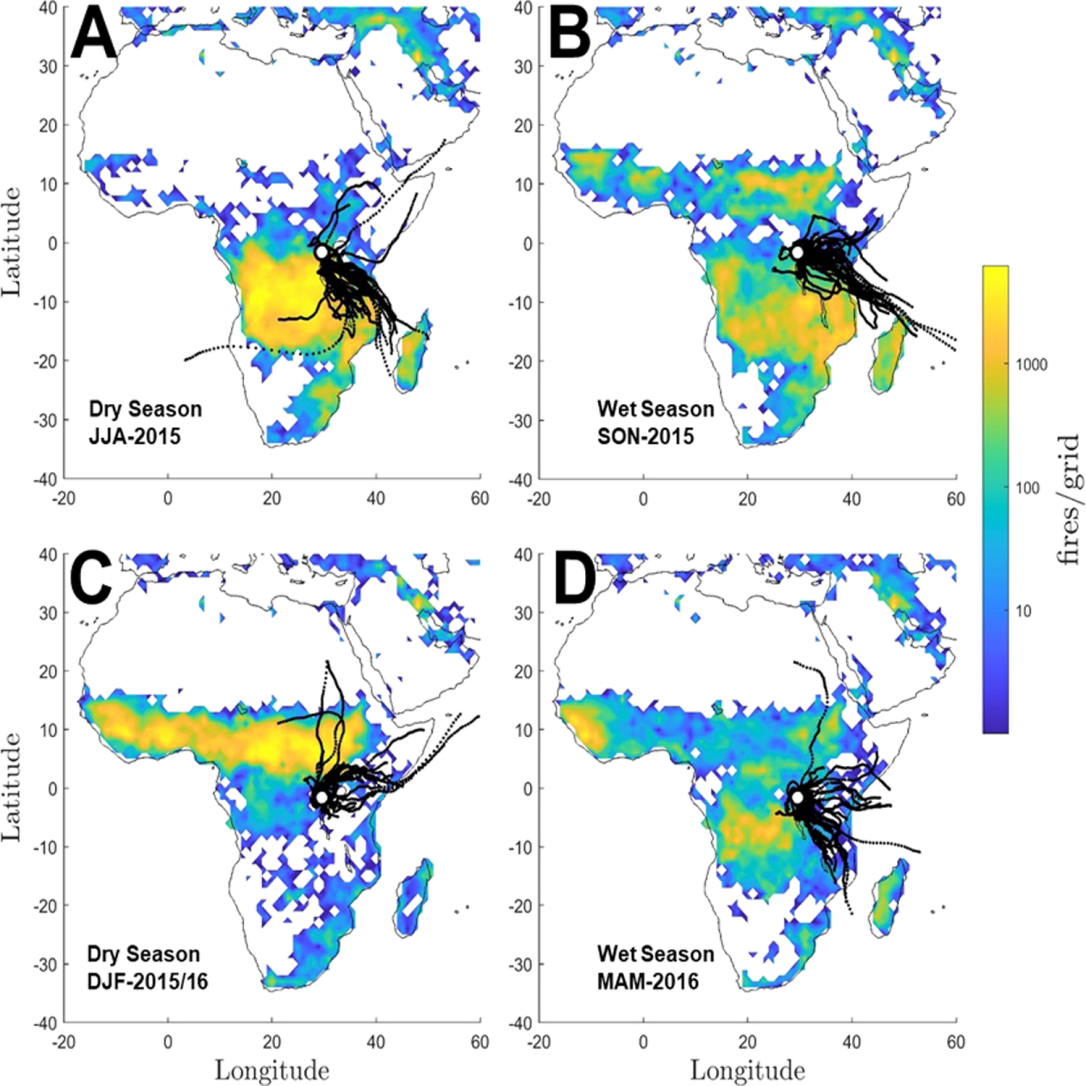
Satellite fire observations and air mass back trajectories at the Rwanda Climate Observatory. The blue-green-yellow color scheme depicts the number of fire detections from MODIS-FIRMS per square degree grid. Every second day, back-trajectory with arrival time at 3 AM CET is depicted as black dotted lines. (A) June–July–August (JJA), 2015. (B) September–October–November (SON), 2015. (C) December–January–February (DJF), 2015/16. (D) March–April–May (MAM), 2016.

Overall, the measured concentrations and aerosol composition are consistent with previous findings in rural and remote locations across sub-Saharan Africa (see compilation by Andersson et al.^1^). The peak aerosol concentrations coincide with upwind regional biomass burning episodes. During the dry boreal winter (December–February), the air masses are largely of northeastern origin and coincide with the northern Sub-Saharan fire band, while air masses are mainly of southeasterly origins during summer (June–August), analogously coinciding with Southern African large-scale fires (Figure 2). This suggests that fire episodes have a substantial impact, while meteorology, e.g., wet scavenging, may also significantly influence aerosol concentrations during monsoon seasons (Figure 1D).

### PM_2.5_ Aerosol Source Regime at RCO

The aerosol chemical composition provides insights into the emission source regime. In this study, BC is well correlated with OC, K^+^, and NO_3_^–^, which indicates a common origin (*R*^2^ > 0.87; *P* < 0.01; SI Figure S5). A linear fit of BC vs both K^+^ and NO_3_^–^ passes through the origin, suggesting a similar source for these three species, likely from biomass emissions (SI Figure S5). In contrast, a nonzero *y*-intercepts are observed when correlating OC with BC (*R*^2^ = 0.87; *y*-intercept = 1.3 μg m^–3^), NO_3_^–^ (*R*^2^ = 0.93; *y*-intercept = 1.9 μg m^–3^), and K^+^ (*R*^2^ = 0.93; *y*-intercept = 1.1; SI Figure S5). This suggests an additional nonbiomass burning background domain of OC, potentially from primary emissions or secondary aerosol formation from biogenic VOC emissions from the forested surroundings.

Overall, we find higher K^+^/BC and NO_3_^–^/BC ratios during the dry periods, contrary to SO_4_^2–^/BC, NH_4_^+^/BC, and OC/BC trends (SI Figure S6). A high OC/EC ratio (seasonal averages >9) is associated with biomass emissions, but is also influenced by atmospheric aging and source variability, e.g., fuel type and burning conditions. While SO_4_^2–^ is associated with savanna fires, it is also elevated from fossil emissions and volcano degassing, suggesting that a mixed source profile may explain the SO_4_^2–^/BC time dependence.^1,42,43^ Overall, the observed mass ratios and correlations are consistent with predominantly biomass burning aerosol emissions, and in sync with the vast regional fires. While local biomass burning influence on our measurements is possible, the high-altitude mountain site is less influenced by the locally influenced planetary boundary layer during the current night-time sampling.^24^

### Multiyear Equivalent BC (eBC) Concentrations

**Figure 3:**
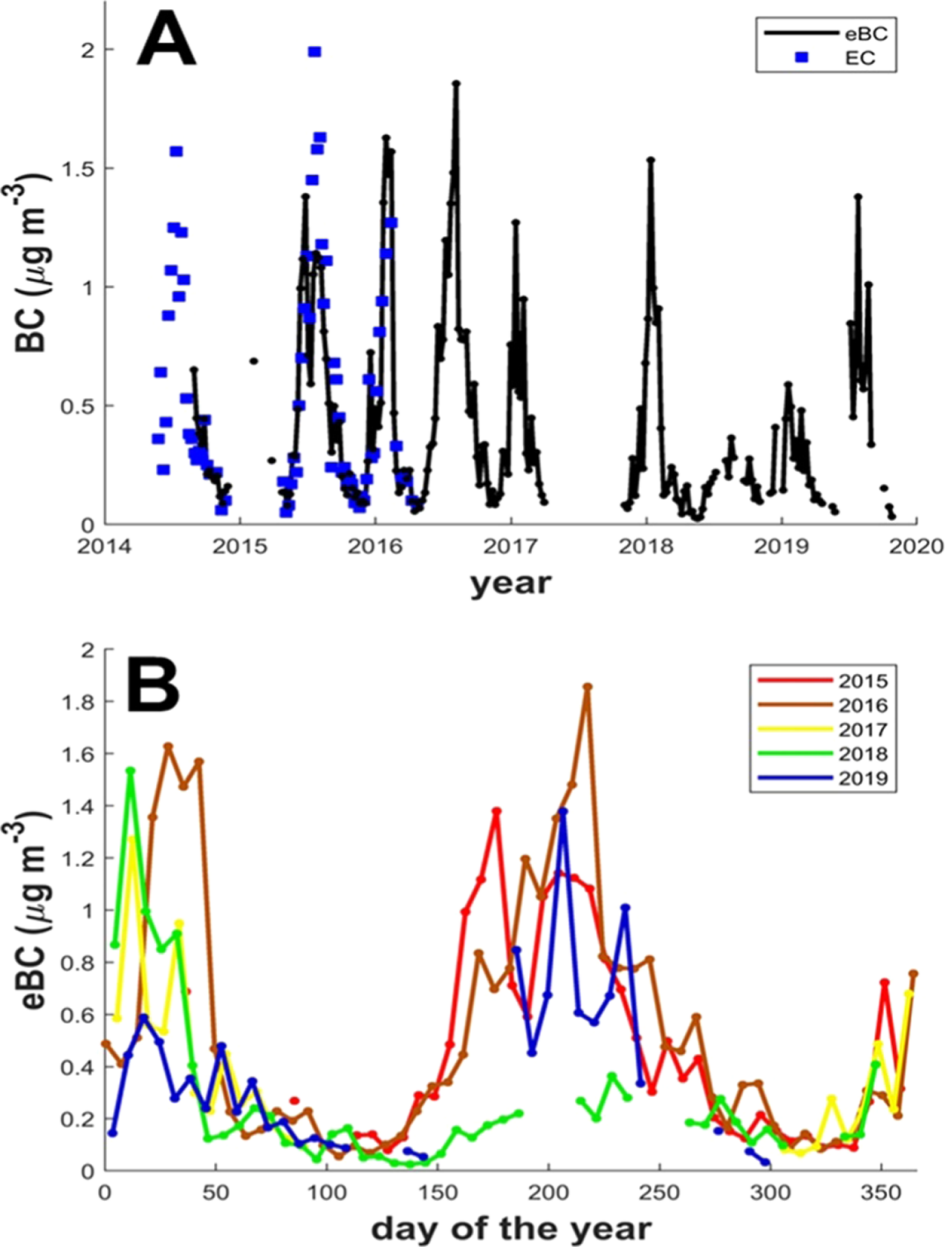
Multiyear (2014–2019) BC concentrations trends for Rwanda Climate Observatory. (A) Temporal variability in weekly averaged (night-time-only) equivalent BC data—retrieved from an AE33 aethalometer at 880 nm. The eBC (black plot) was compared against the Sunset Laboratory thermo-optical measurement data (blue dots; see SI notes S1). (B) Visualization of differences in daily/weekly de-trended annual variability in eBC concentrations (color scheme depicts different years). The spikes in the aethalometer data, potentially from instrumental errors or short-term pollution events were removed as explained in SI Note S1.

Multiyear (2014–2019) aethalometer eBC data mirrors the seasonal oscillations observed in carbonaceous aerosols and inorganic ions (Figures 2 and 3). Concentration-weighted back-trajectory analysis shows that the eBC concentrations are elevated when the air masses are from the north and from the south, overlapping with the large-scale fires occurring during the dry seasons (SI Figure S7). A notable interannual variability is also observed when comparing the seasonal trend for different years (Figure 3B). Such changes may be attributed to many factors, e.g., the spatio-temporal interplay with air mass transport and fires or rainfall. In addition, large-scale climatological phenomena may modulate the fire regime, e.g., the El Nino Southern Oscillation, which had a maximum during 2015, resulting in drier regions in Southern Africa and a wetter one in the Eastern Africa region.^44^ Overall, the BC concentrations and seasonal cycles mirror the large-scale dynamics of the Eastern Africa savanna fire emissions, as influenced by the region’s meteorology.

### Isotope-Based Source Quantification of BC

**Figure 4:**
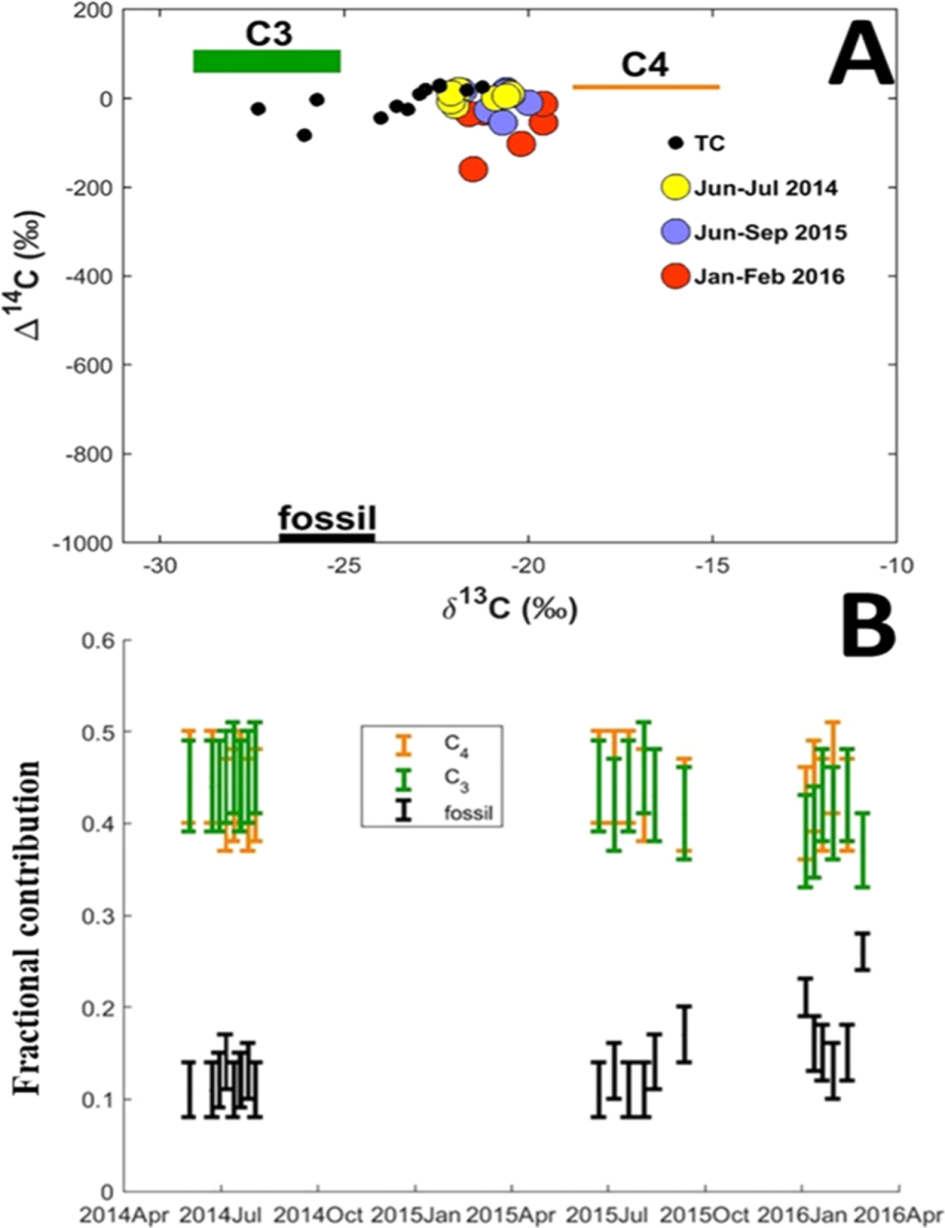
Dual-carbon (Δ^14^C and δ^13^C)-based source apportionment of BC at Rwanda Climate Observatory. (A) Dual-isotope signatures of BC for dry period PM_2.5_ aerosols. Colored circles represent the isotope signatures for BC for the study period (color represents time period), while black dots represent the isotope signatures for total carbon (TC = EC + OC) from October to November 2014 and May to September 2015.^1^ (B) Relative source contributions (means and standard deviations), computed using eq 2.

Dual-carbon isotope (Δ^14^C and δ^13^C) signatures offer a high-precision approach to quantify the main source contributions to BC in the SSA atmosphere. Overall, the Δ^14^C signatures observed here, ranging between −159 and +18‰, signal a strong biomass burning influence (Figure 4). The corresponding δ^13^C signatures were determined to be within a narrow range of −20.9 ± 0.8‰, suggesting minimal variation within the source fractions. While the Δ^14^C signatures are comparable to those previously reported at RCO but for the bulk carbonaceous aerosols (total carbon), the observed δ^13^C-BC signatures are enriched by ∼2‰ in ^13^C (Figure 4A).^1^ Unlike OC, δ^13^C-BC exhibit minimal shifts during atmospheric aging.^1,43^

The Δ^14^C signatures unambiguously reveal very high biomass burning contributions (93 ± 3%) to BC at this SSA regional background environment during the dry seasons. The findings differ from those in a previous absorption Ångström exponent (AAE)-based assessment, where over 50% of dry period BC at RCO was assigned to the fossil fraction.^24^ The AAE method is semiquantitative and more accurate if calibrated to local conditions, e.g., burning conditions, fuel mix, and aerosol aging, while the standard model and end members used in that study were based on studies conducted elsewhere.^24,45^ The Δ^14^C signatures show a higher fossil fraction, up to 20%, is realized during the dry December–February period. However, the interseasonal BC loadings from fossil sources are comparably low and near-constant (140 ± 40 ng m^–3^), while the corresponding contribution from biomass burning (BC_bio_ = *f*_bio_·BC) is much larger and more variable (970 ± 210 ng m^–3^; SI Figure S8). This suggests that the BC_fossil_ loadings during the dry periods represent a background regime, while the BC_bio_ is more influenced by events from long-range transport of air masses.

The relationship between the isotopic signatures and the BC concentration, following the Keeling plot approach, can give insights into the emission source profile. A linear fit between the Δ^14^C-BC signatures with the inverse of the total BC concentrations (1/BC; *R*^2^ = 0.71; *P* < 0.05; SI Figure S9) suggests that the BC loadings may be described as a two-state mixture; a stable background and a large variable input.^46^ A similar relation was found for total carbon (TC = OC+TC) at RCO for an overlapping period, 2014/15.^1^ The Δ^14^C signatures, in line with the above argument, show that the background regime is enriched in fossil contributions. The Δ^14^C value along the linear fit where 1/BC → 0 (BC→∞) provides information regarding the isotopic signature of the large, temporally varying source, which here is Δ^14^C = +57 ± 13‰. This is indeed also the estimated average for the biomass burning endmember (+57 ± 52‰).^1^ This suggests two things: 1. The present biomass burning endmember is coherent with the observational data; 2. The temporally varying source is almost 100% of biomass burning origins.

Combining the Δ^14^C and δ^13^C signatures allows the separation of the estimated fraction of biomass burning into two fractions: burning of C3 plants (e.g., woody plants) and burning of C4 plants (eq 2). Bayesian source apportionment reveals that the relative contributions of C3 plants and C4 plants to BC are overlapping and near-equal (*f*_C4_/(*f*_C4_ + *f*_C3_) = 50 ± 1%) for all investigated samples (Figure 4B). This indicates that the composition of the temporally varying large biomass source is stable over time, despite the air masses during the two dry periods reflecting quite different geographical regimes (Figure 2). C4 plants are abundant savanna biomass but are almost depleted in forests. The estimated fractional contribution from C4 plants to the biomass in African savannas is quite variable (34–71%).^47^ Here, the small variability suggests that this is mainly from one source type, as combinations of different sources are expected to increase variability. This assessment is corroborated by the elevated correlations between BC, K^+^, and NO_3_^–^, which combined form a savanna-specific source marker. Taken together, we conclude that the biomass burning activities that influence BC in the SSA region during dry periods are near-exclusively from savanna fires.

### Top-Down Observational Constraints on the Relative Emission Factors

Emission factors, calculated as the amount of material emitted per ton of fuel burned, are fundamental, but highly uncertain in emission inventories. This uncertainty is especially high for the biomass fraction.^2,6^ Reasons for its large variability include the large number of different conditions under which a material may burn (e.g., flaming or smoldering fires) and the variability of the fuel (e.g., different types of hard or softwoods, grasses, and water contents). However, concentrations in the atmosphere do not mirror this large variability. A key reason is that the variability is suppressed when many different emissions combine, following the central limit theorem.^36^ Since top-down techniques involve conducting measurements on the mixed signal, one therefore expects less variable estimates.

Analysis carried out in this study—aerosol composition, correlations, and isotopic source constraints—strongly suggest that BC aerosols in the SSA background atmosphere are almost exclusively modulated by savanna fires. Furthermore, the significant correlations between OC, BC, K^+^, and NO_3_^–^ suggest that, while they may be differently affected by atmospheric processing (e.g., photochemistry and cloud interactions), the source relations are largely preserved during atmospheric transport. Emissions are determined by the product of the activity (amount of fuel burned) and the emission factor. Given the shared origins of these components (OC, BC, K^+^, and NO_3_^–^), the emission factor is thus the main variable in emission estimates. Therefore, by examining the slope of the different components, we may have the means to approach effective tropospheric relative emission factors (EF), by which we here mean to be the ratio of the emission factor of one component (X) relative to a reference, here BC (EF_X_/EF_BC_).

The slopes of OC/BC, K^+^/BC, and NO_3_^–^/BC are 6.9 ± 0.3, 0.85 ± 0.04, and 1.60 ± 0.06, respectively (for error propagation, see SI Note S3). Meanwhile, a compilation of bottom-up emission factors from savanna fires (by Andreae, 2019) gives: EF_OC_/EF_BC_ = 5.7 ± 4.7, EF_k+_/EF_BC_ = 0.8 ± 0.7, and EF_NOx_/EF_BC_ = 4.7 ± 4.0 (variability of ratios was calculated using error propagation from published data).^2^ First, we note that for OC, BC, and K^+^, the EF ratios and the slopes are largely overlapping; however, the variability in the top-down estimates (the slopes) is much lower. Second, there are no emission factors for NO_3_^–^, but only for NO_x_, while NO_x_ is a precursor for NO_3_^–^ in the atmosphere. While the numbers are therefore not directly comparable, the current estimate provides information for parametrizing satellite-based NO_3_^–^ emissions estimates. Taken together, and given the underlying assumptions of this argument, these observational results constitute better constrained relative emission factors for OC/BC, K^+^/BC, and NO_3_^–^/BC for large-scale emissions from savanna fires in Africa, with applications to parametrizations of bottom-up and satellite-based emission inventories.

### Scientific and Policy Implications

The fractional source contributions of biomass burning to BC in the sub-Saharan African background atmosphere are here constrained to be as high as 95%, which is higher than what is observed using the same isotope-based methodology at remote sites in South Asia (∼50%),^20,48−50^ Southeast Asia (∼70%),^50^ East Asia (∼30%),^27,51−53^ Europe (∼30%),^54,55^ the Tibetan Plateau and Himalayas (∼50%),^56^ and the Arctic (∼40%).^22,30,33^ Therefore, SSA is not only a very high-emitting region but also has a very different aerosol regime compared to most other locations around the globe.

Overall this emphasizes the need to further investigate this region, as knowledge about BC—and aerosols in general—obtained from other regions may not be transferable to this region.^24^ This has implications for satellite-based and bottom-up estimates of BC emissions from large-scale fires: the parametrizations (e.g., emission factors) from other regions are unlikely to apply in this region.^5^ Furthermore, there is a need to fine-tune chemical transport and climate models with region-specific parametrizations, as well as the need for continuous and expanded ground-based observations of aerosols in Africa.^12,23^

Organic mass, the dominant aerosol component—in addition to BC—need to be further investigated, including the light-absorbing fraction—brown carbon (BrC). The slash-and-burn agricultural practices and high household reliance on biofuels are some of the policy target areas, as well as the growing fossil BC emissions in African cities.^5,18^ Overall, this study stresses the need to further constrain the uncertainties regarding the impact of aerosols on the warming climate in Africa.

## Supporting Information Available

Discussions on analysis of aethalometer data (Note S1); calculation of sea salt contribution (Note S2) and error propagation (Note S3); quality assurance data (Table S1); sensitivity analysis for isotope measurements (Table S2); seasonal averaged PM_2.5_ aerosol composition (Table S3); BC loading and sample selection for isotope analysis (Figure S1); equivalent BC data and quality assurance (Figures S2, S3, S10, and S11); PM_2.5_ aerosol composition and correlations (Figures S4–S6); concentration-dependent geographical origins of BC (Figure S7); and isotope-based source apportionment of BC (Figures S8 and S9); observational data that support the findings of this study will be available in the Bolin Centre Database (bolin.su.se/data/) (PDF)
