# Supplementary material for: Atmospheric Black Carbon Loadings and Sources over Eastern Sub-Saharan Africa Are Governed by the Regional Savanna Fires
Source: Environ Sci Technol. 2022 Oct 30;56(22):15460–9. doi: 10.1021/acs.est.2c05837 (PMC9670846; doi:10.1021/acs.est.2c05837)
Supplement: Supplementary file 1 — es2c05837_si_001.pdf [file es2c05837_si_001.pdf]

Supporting Information

Atmospheric Black Carbon Loadings and Sources over Eastern Sub-Saharan Africa are Governed by the Regional Savanna Fires

Leonard Kirago[1,2], Örjan Gustafsson[1,2], Samuel M. Gaita[1,2], Sophie L. Haslett[1,2], H. Langley deWitt[3,a], Jimmy Gasore[3,4,5], Katherine E. Potter[3,b], Ronald G. Prinn[3], Maheswar Rupakheti[6], Jean de Dieu Ndikubwimana[4], Bonfils Safari[5], and August Andersson[1,2*].

[1]Department of Environmental Science, Stockholm University, 10691 Stockholm, Sweden

[2]Bolin Centre for Climate Research, Stockholm University, 10691 Stockholm, Sweden

[3]Center for Global Change Science, Massachusetts Institute of Technology, 54-1312 Cambridge, Massachusetts, USA

[4]Climate Secretariat, Ministry of Education, 622 Kigali, Rwanda

[5]Physics Department, School of Physics, College of Science and Technology, University of Rwanda, 4285 Kigali, Rwanda

[6]Institute for Advanced Sustainability Studies (IASS), 14467 Potsdam, Germany

[a]now at: Cooperative Institute for Research in Environmental Sciences, University of Colorado at Boulder, 80309 Colorado, USA.

[b]now at: Boulder A.I.R. (Atmosphere Innovation Research) LLC, Boulder, 80305 Colorado, USA

***Correspondence:** August Andersson (august.r.andersson@gmail.com)

Supproting information includes: 18 pages; 3 Notes, 3 Tables, and 11 Figures.

Contents

Supporting Notes......3
SI Notes S1: Analysis of Aethalometer Data......3
SI Notes S2: Non-sea salt contribution to aerosol concentration 4
SI Notes S3: Variability of the slope in linear regression......4
Supporting Tables......5
SI Table S1: Quality assurance data for analytical methods used. 5
SI Table S2: Sensitivity analysis for inadvert inclusion of pyrolyzed carbon into EC fraction. 5
SI Table S3: $PM_{2.5}$ aerosol composition, mass ratios, and dual carbon isotopes......6
Supporting Figures 7
SI Figure S1: BC loadings and sample selection for carbon isotopes analysis. 7
SI Figure S2: Hourly and weekly averaged equivalent BC data. 8
SI Figure S3: Histograms of de-trended eBC showing a log-normal distribution......9
SI Figure S4: Seasonal changes in $PM_{2.5}$ aerosol composition at the RCO......10
SI Figure S5: Correlation between different $PM_{2.5}$ components at RCO. 11
SI Figure S6: Temporal trend of different mass ratios relative to BC in PM2.5 aerosols......12
SI Figure S7: Visualization of concentration-dependent geographical origins of BC......13
SI Figure S8: Dual-carbon isotope-based BC source attribution at RCO......14
SI Figure S9: The dual carbon isotope signatures of BC, and the respective keeling plots. 15
SI Figure S10: Spike-detection of the hourly resolution eBC data......16
SI Figure S11: Correlation between eBC estimates and thermo-optical BC data. 17
References......18

Supporting Notes

SI Notes S1: Analysis of Aethalometer Data

High temporal resolution (hourly) equivalent BC (eBC) data was retrieved from an AE33 aethalometer at 880nm (Magee Inc.). The 1 to 5 minutes resolution aethalometer data were binned into an hourly resolution format (median). Spikes in the hourly data were removed by the following sliding window algorithm: i) the mean (μ) and standard deviation (□) of four adjacent data points (i-2; i-1; i+1 and i+2) around data point i is calculated. ii) If the value at point i is higher than μ + 7·□ (positive spike) or is lower than μ - 7·□ (negative spike), then this data point is considered a spike and is removed (Figure S10). Also, data points with fewer than 2 adjacent data points were removed. Out of in total 30015 hourly data points, 187 were removed, corresponding to 4‰ (the number 7 was chosen such that less than 5‰ of the data should be removed). Positive spikes were over-represented during 6 - 7 PM, local time, suggesting influence by, e.g., evening cooking/heating.

The aethalometer estimates the BC concentrations by measuring the attenuation of light through a filter tape. Calculation of BC concentration estimates requires three transformations of the raw data: i) accounting for the non-linear light-attenuation in a filter; ii) correction for multiple scattering (MS) effects; iii) conversion of the correct light-attenuation signal (m-1) to BC concentrations ($\mu g\ m^{-3}$) by a conversion factor (mass absorption cross-section, MAC). Here, we used the method of Drinovec et al.,[1] for these three steps. Both the MS and the MAC, in general, depend on the state of the particles, including a degree of internal/external mixing and/or interference between BC and other absorbing components, e.g., brown carbon. These effects have been shown to be highly site-specific. The MS may vary from ~ 2 for fresh BC to up to ~ 6 for biomass burning influenced BC in the Amazon [2,3], while the MAC may be enhanced by a factor of 2 or more due to coatings [4]. For practical purposes of estimating BC concentrations (as opposed to estimating light-absorption coefficients), the distinction between MAC and MS is less important, as we can consider an effective correction factor X = MAC/MS.

Given the site-specific dependency on the highly variable factor X, we here calibrate the BC estimates from the aethalometer against sunset laboratory thermo-optical measurement data. The hourly resolved aethalometer data was matched to the week-long (night-time only) filter collections. Linear regression of the 55 overlapping data points gives a correlation of $R^2 = 0.83$ ($p<<0.01$), suggesting fair agreement (Figure S11). Overall, the BC concentrations were found to be over-estimated by a factor of 3.2 at 880nm. Similar offsets have been observed previously

for aged biomass burning plumes.[5] A possible explanation is enhancement due to mixing state in the aged air masses arriving at RCO.

SI Notes S2: Non-sea salt contribution to aerosol concentration

The non-sea-salt (nss) concentrations of K^+ and SO_4^{2-} were calculated using Na^+ as the sea-salt tracer [6,7].

nss-SO_4^{2-} is computed as follows:

$$nss\ SO_4^{2-} = [SO_4^{2-}] - \left[\left(\frac{[SO_4^{2-}]}{[Na]}\right)_{\text{sea}} * [Na^+]\right]$$

where ($[SO_4^{2-}]$/ $[Na^+]$) sea is the standard ratio of the concentrations of SO_4^{2-} and Na^+ which is obtained from seawater composition.[7]

SI Notes S3: Variability of the slope in linear regression

Consider a linear regression of parameters x and y:

$$y = \beta \cdot x + \alpha$$

Where, β is the fitted slope and α is the fitted intercept. The variance (squared standard deviation, σ^2) of the fitted slope may be calculated as:

$$\sigma^2 = \frac{\sum_{i=1}^{n}(y_i - (\beta \cdot x_i + \alpha))^2}{(n-2)\sum_{i=1}^{n}(x_i - \bar{x})^2}$$

Where, n is the number of data points and $\bar{x}$ is the mean of x.

Supporting Tables

SI Table S1: Quality assurance data for analytical methods applied in this study. Method detection limits (MDL = mean + 3*SD of the blanks), field blank contribution, and measurement precision were evaluated for BC, OC and water-soluble inorganics. Units in µg ml^{-1} or µg cm^{-2}.

	**BC**	**OC**	**SO_4^{2-}**	**NO_3^-**	**Cl-**	**NH_4^+**	**Na^+**	**K^+**	**Ca^{2+}**	**Mg^{2+}**
Blank mean	< 0.02	0,45	0.13	0.05	0.06	0.25	0.04	0.02	0.08	0.01
Method Detection limit	0.02	1.12	0.23	0.19	0.08	0.25	0.12	0.07	0.12	0.05
Mean Blank contribution	N.D	4 %	2 %	7 %	48 %	6 %	14 %	3 %	66 %	35 %

ND - not detected

SI Table S2: Sensitivity analysis for inadvert inclusion of pyrolyzed carbon (pryC) into the cyro-trapped EC fraction. Based on Andersson et al. (2020), the $\Delta^{14}C$ for PyrC (TC) at RCO is estimated as +37‰ and $\Delta^{14}C$-BC at -32‰.

**%PyrC in EC**	**$\Delta^{14}C_{BC}^{Obs}$ (‰)**	**$\Delta^{14}C_{OC}^{Obs}$ (‰)**	**$\Delta^{14}C_{BC}^{real}$ (‰)**	**f_{bio}^{real} (%)**
0%	-32	37	-32	0.92
5%	-32	37	-35	0.91
10%	-32	37	-39	0.91
20%	-32	37	-49	0.90
30%	-32	37	-61	0.89

SI Table S3: Seasonal averaged $PM_{2.5}$ aerosol composition (concentrations in µg m^{-3}), mass ratios, and dual carbon isotopes (□^{13}C and $\Delta^{14}C$; ‰) at a sub-Saharan Africa regional background site, Rwanda Climate observatory. The June - August (JJA) and December to February (DJF) period typically have dry weather.

	**OC**	**EC**	**NH4+**	**K+**	**SO_4^{2-}**	**NO_3^-**	**OC/BC**	**NH_4^+/BC**	**SO_4^{2-}/BC**	**K^+/BC**	**NO_3^-/BC**	**□^{13}C**	**$\Delta^{14}C$**
**JJA, 2014**	7.9 ± 3.0	0.7 ± 0.4	1.1 ± 0.5	0.8 ± 0.3	2.5 ± 1.2	1.3 ± 0.7	11 ±3	1.7 ± 0.7	3.8 ± 1.9	1.2 ± 0.3	1.7 ± 0.6	-21.3 ± 0.8	+3.3 ± 11
**SON, 2014**	2.6 ± 1.1	0.2 ± 0.1	0.5 ± 0.3	0.2 ± 0.1	1.3 ± 0.8	0.2 ± 0.1	12 ± 3	2.4 ± 0.9	5.9 ± 2.5	0.8 ± 0.2	0.5 ± 0.4		
**JJA, 2015**	8.6 ± 2.8	1.1 ± 0.5	1.1 ± 0.4	0.9 ± 0.3	2.2 ±1.1	1.5 ± 0.7	9 ± 3.2	1.2 ± 0.9	2.7 ± 2.7	0.8 ± 0.2	1.3 ± 0.3	-20.8 ± 0.6	-7.7 ± 29
**SON, 2015**	2.5 ± 1.6	0.3 ± 0.2	0.4 ± 0.2	0.2 ± 0.2	1.0 ± 0.6	0.2 ± 0.2	12 ±4	1.8 ± 0.7	4.8 ± 1.8	0.7 ± 0.4	0.7 ± 0.3		
**DJF, 2015/16**	5.7 ± 3.5	0.6 ± 0.4	0.9 ± 0.6	0.7 ± 0.5	2.4 ± 1.3	0.9 ± 0.8	9 ±1.6	1.5 ± 0.5	3.9 ± 1.1	1.0 ± 0.2	1.1 ± 0.6	-20.6 ± 0.9	-66 ± 55
**MAM, 2016**	2.6 ± 1.1	0.2 ± 0.1	0.4 ± 0.2	0.1 ±0.1	1.0 ±0.61	0.2 ± 0.1	18 ± 4	2.5 ± 1.1	6.5 ± 3.5	0.7 ± 0.4	0.8 ± 0.5		

Supporting Figures

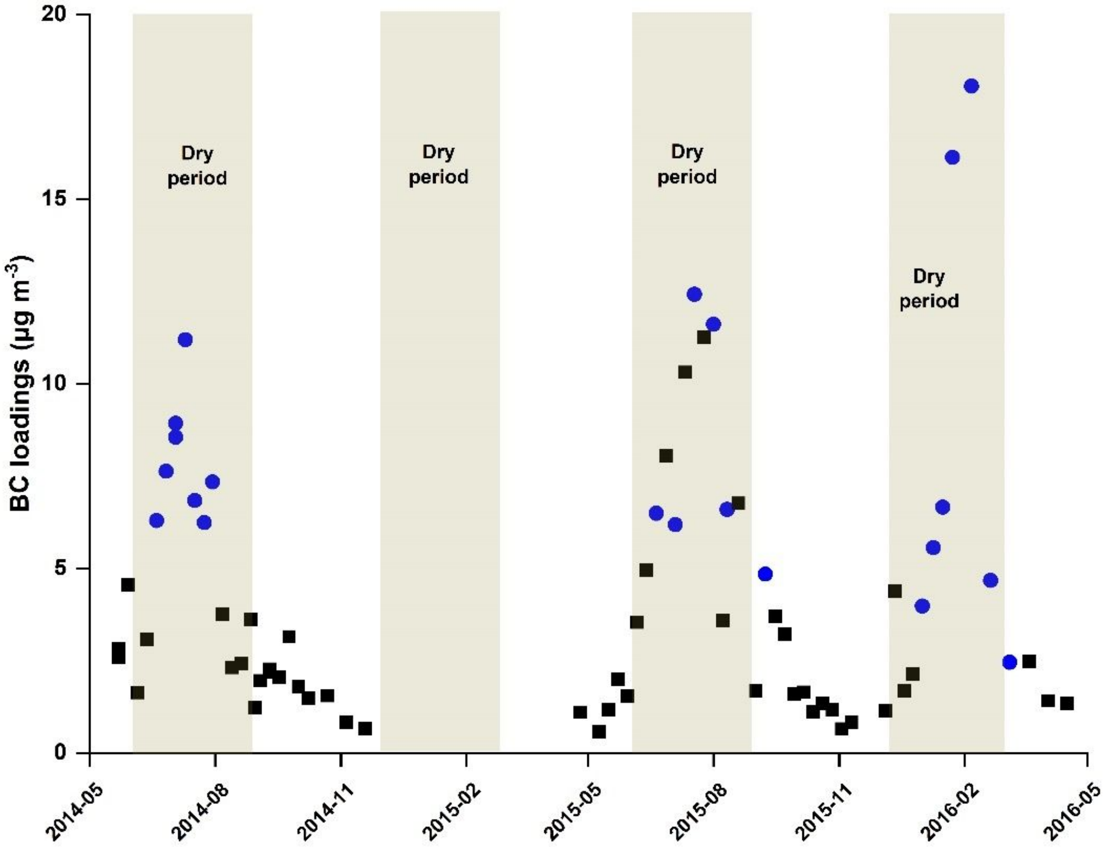

SI Figure S1: Sample filter BC loadings – using sunset laboratory thermo-optical instrument - and samples selected for carbon isotopes analyses. Blue circles represent the selected samples for isotope analyses, mainly during the highly BC-loaded dry period (highlighted in greyish colour). Data gap exists between December 2014 and April 2015 due to instrument failure following a lightning strike.

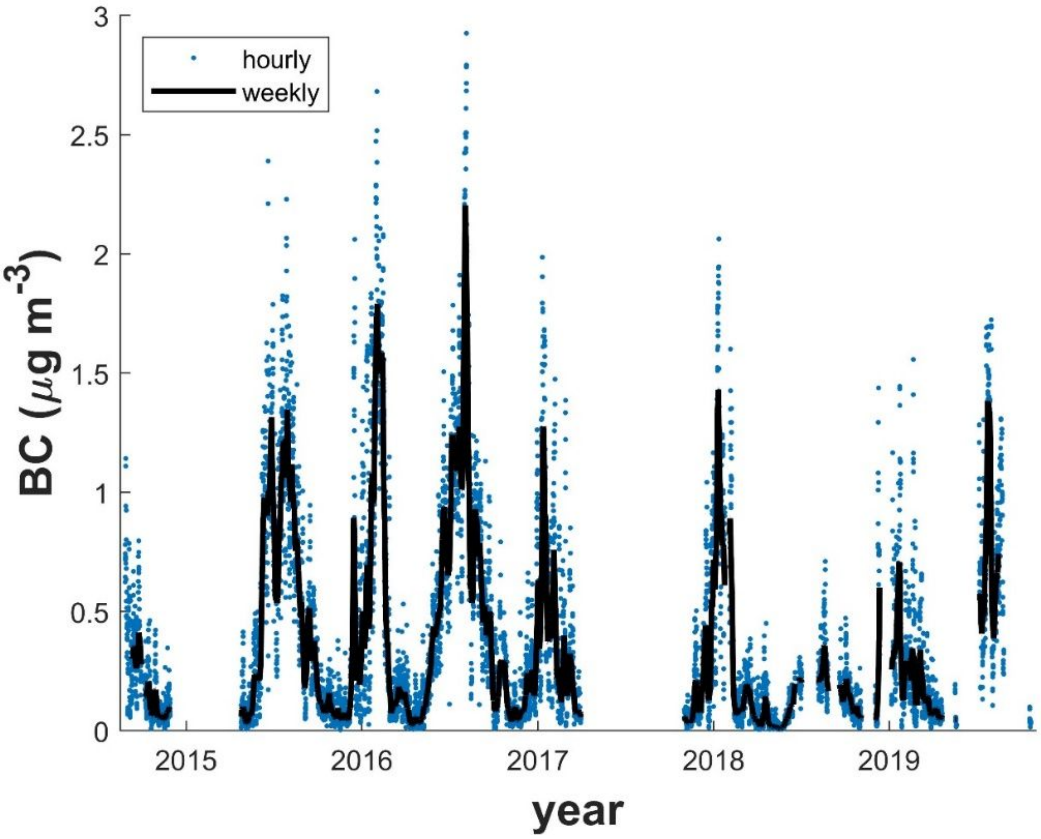

SI Figure S2: Hourly (blue dots) and weekly (black trendline) averaged equivalent BC data from an AE33 aethalometer at 880nm. The spikes in the hourly resolution data, potentially from short-term pollution events, were removed following the sliding window algorithm.

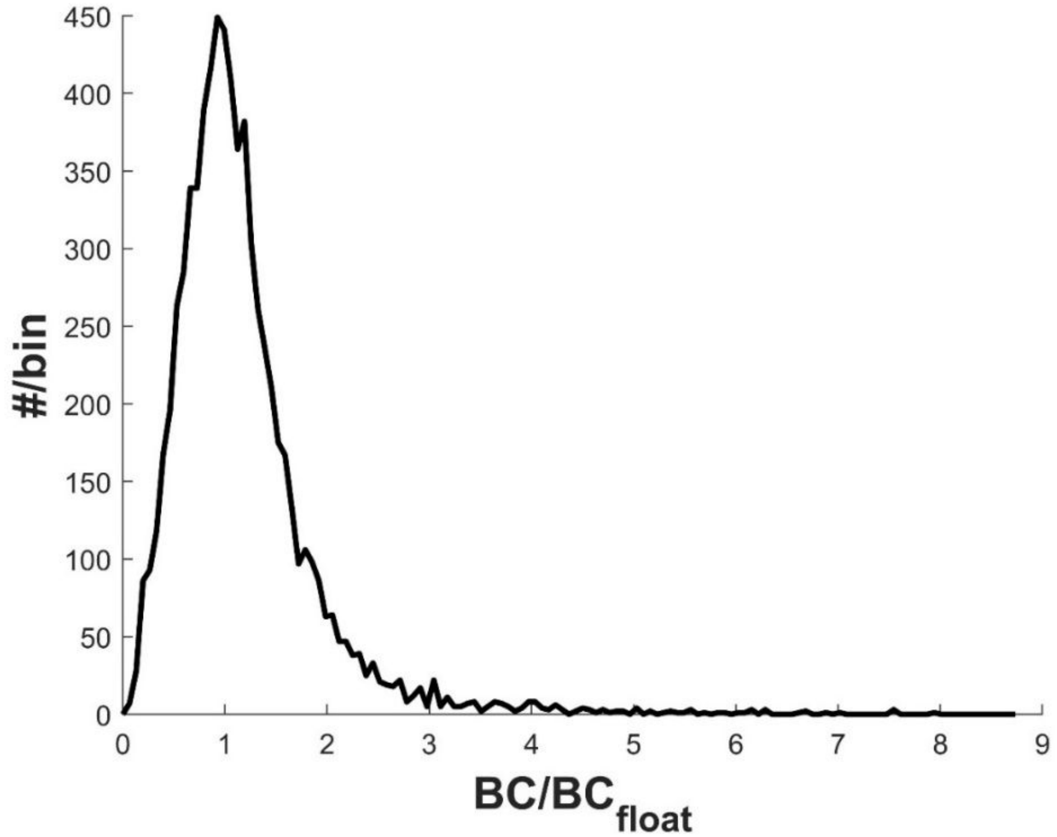

SI Figure S3: Histograms of de-trended equivalent BC data (eBC; hourly data divided by weekly floating averages) showing a log-normal concentration distribution. High temporal resolution eBC data was retrieved from an AE33 aethalometer at 880nm.

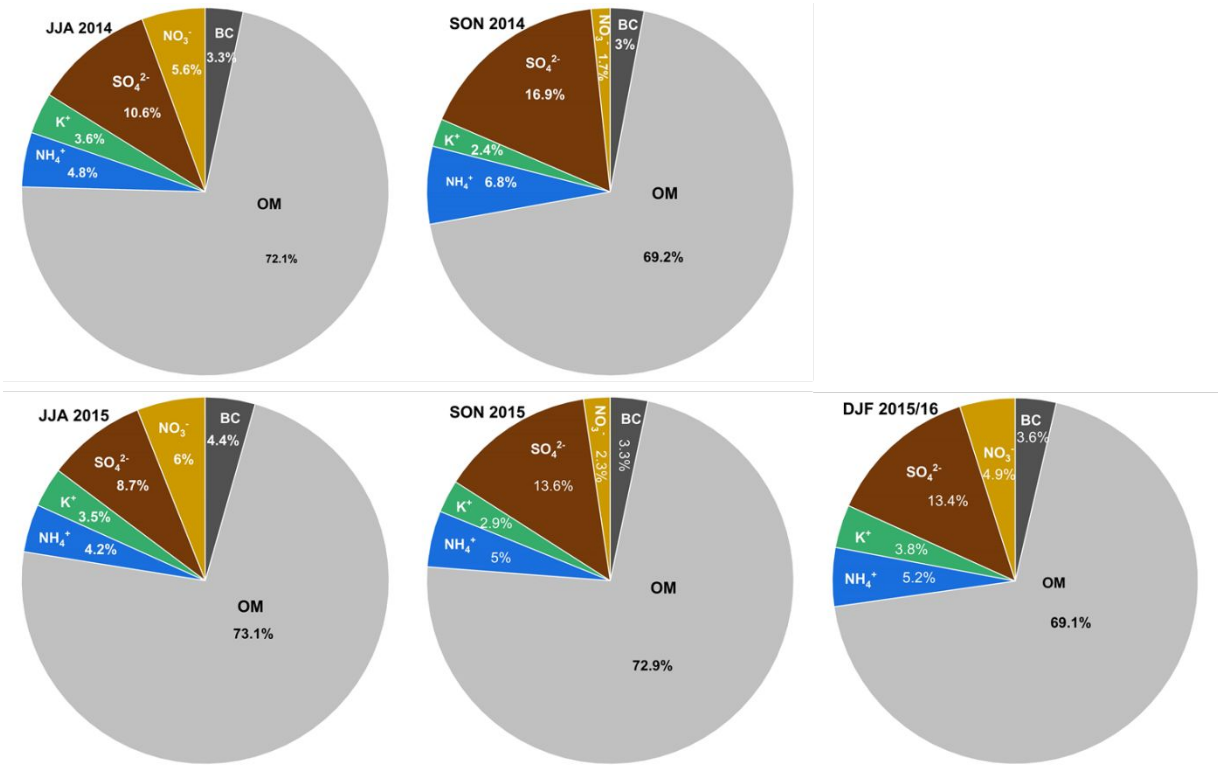

SI Figure S4: Seasonal changes in $PM_{2.5}$ aerosol composition at the Rwanda climate observatory. June-July-August (JJA) and December-January-February (DJF) are classified as dry periods, while a wet season is experienced between September and November (SON.)

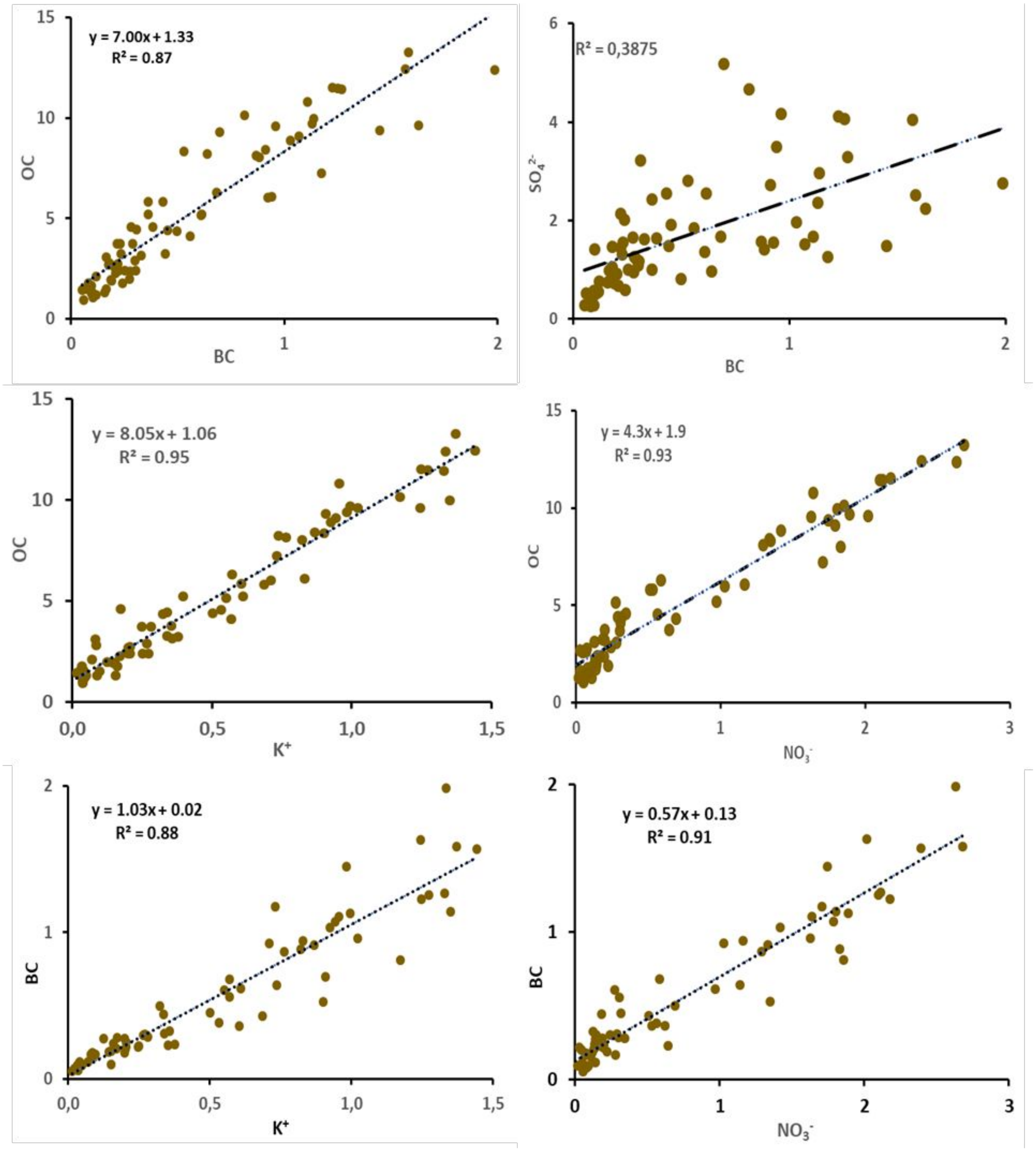

SI Figure S5: Correlation between different $PM_{2.5}$ components in aerosol samples collected at Rwanda Climate Observatory. Highly correlated BC, OC, NO_3^- and K^+, suggest a common emission source profile.

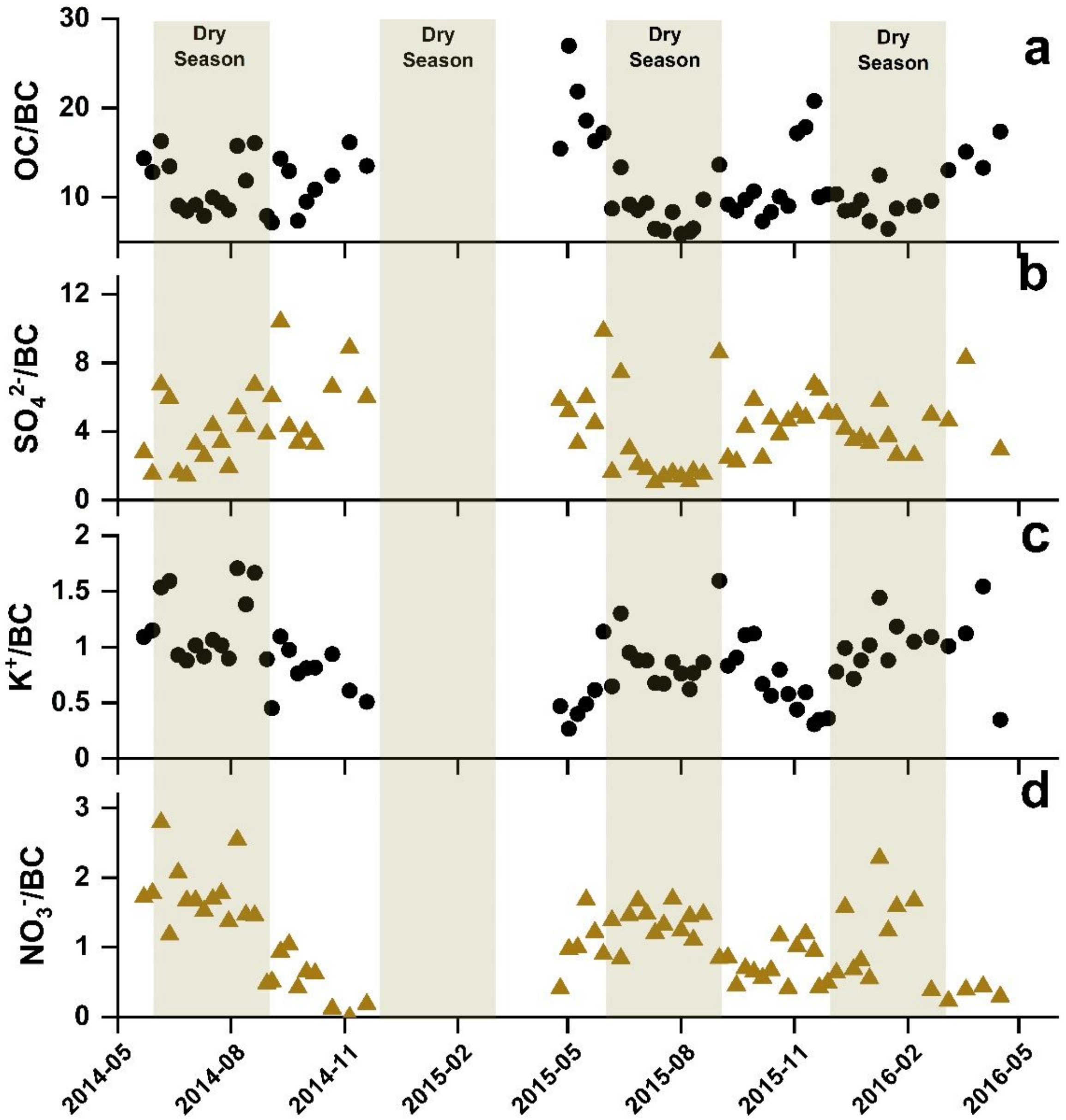

SI Figure S6: Temporal trend of different mass ratios relative to BC in PM2.5 aerosols collected at the Rwanda Climate Observatory between May 2014 and June 2016. Panel (a) shows the OC/BC, panel (b) shows the SO_4^{2-}/BC, panel (c) shows the K^+/BC, and panel (d) shows the NO_3^-/BC trend. Data gap exists between December 2014 and April 2015 due to instrument failure after a lightning strike. The dry periods are highlighted.

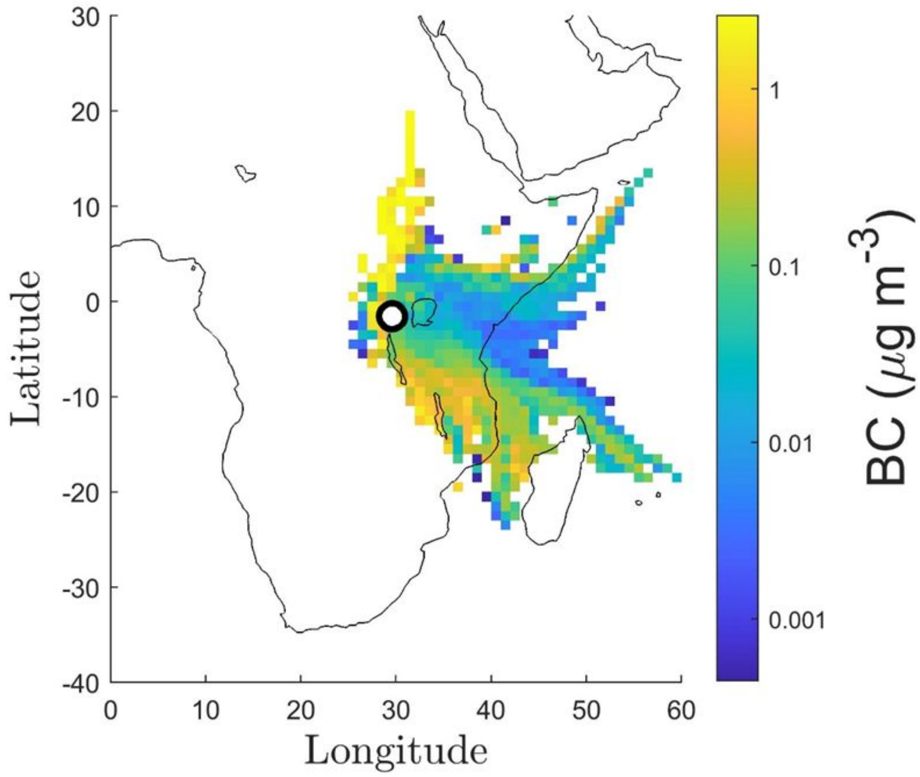

SI Figure S7: Visualization of concentration-dependent geographical origins of Black carbon. Black carbon is predominantly of Northeastern origin between December to February, and SouthEastern origin during Boreal summer, overlapping with largescale savanna fire occurrence.

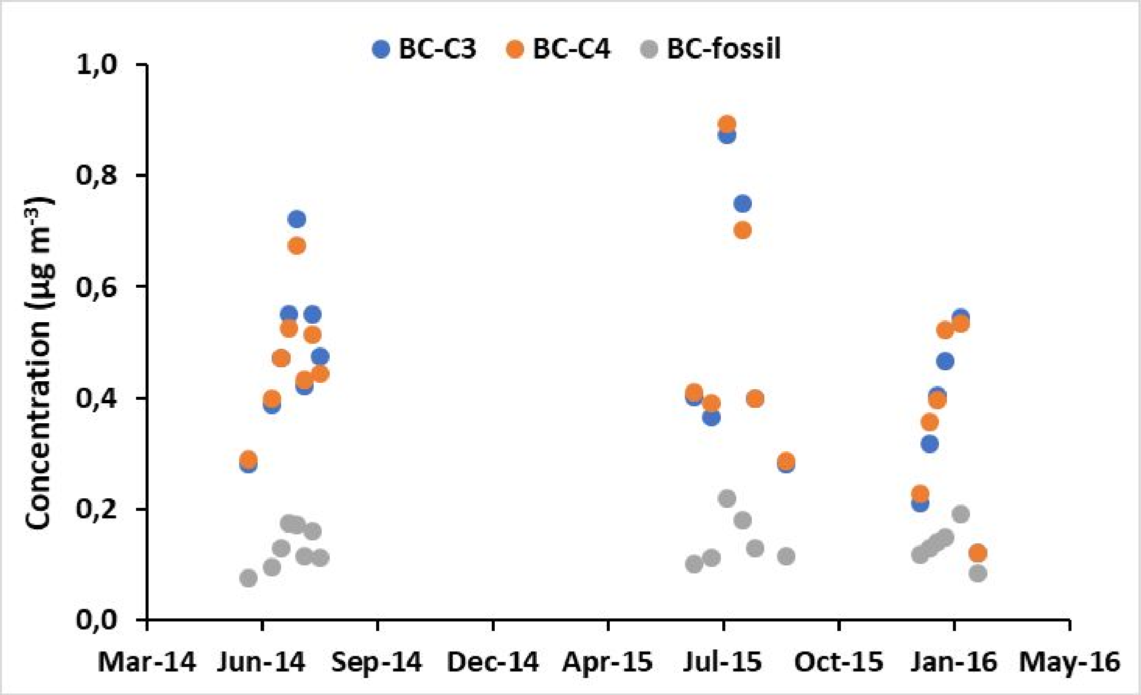

SI Figure S8: Dual-carbon (Δ^{14}C and □^{13}C) isotope-based BC source attribution at Rwanda Climate Observatory. The main sources for carbonaceous aerosols in Eastern Africa are C3 plants, C4 plants, and liquid fossil combustion. The source fractions and their uncertainties were estimated through Markov chain Monte Carlo (MCMC) simulations.

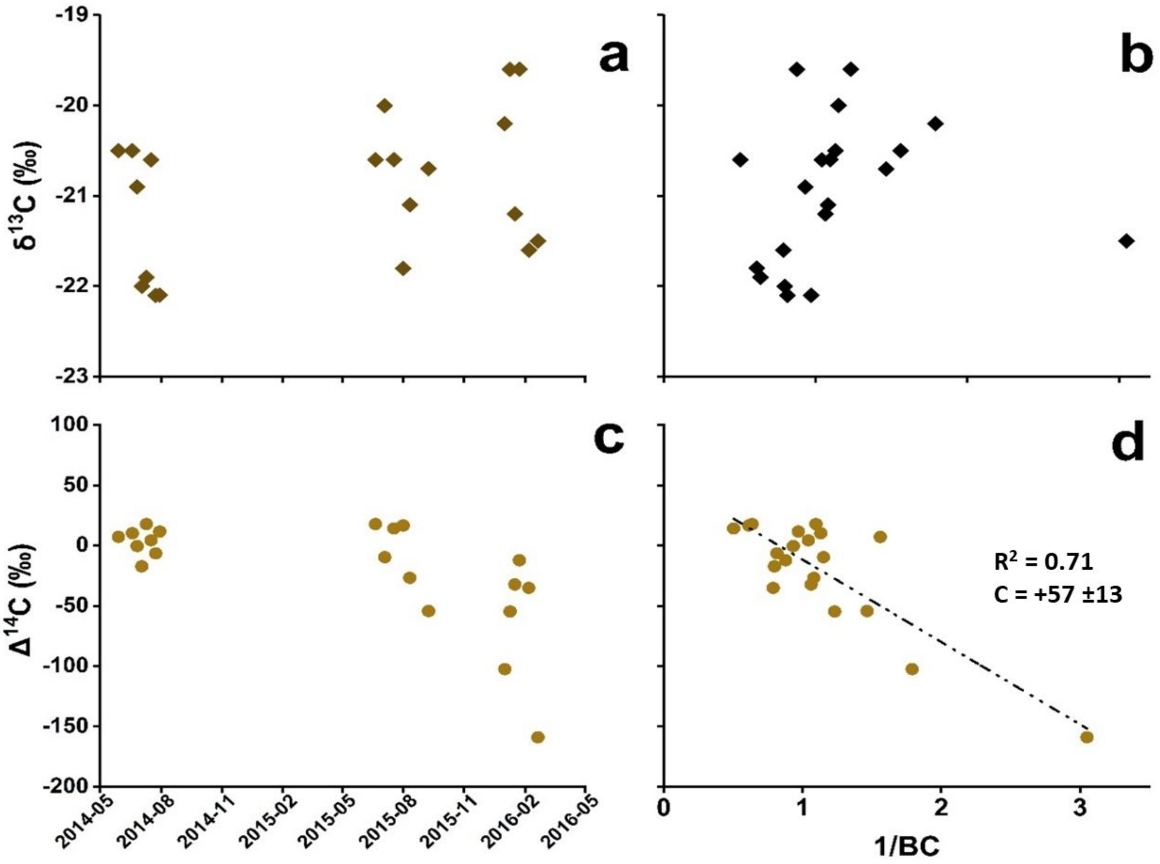

SI Figure S9: The dual carbon isotope signatures ($\Delta^{14}C$ and $\delta^{13}C$) of BC, and the respective keeling plots. The time-series for the observed $\delta^{13}C_{BC}$ signatures (a) and $\Delta^{14}C_{BC}$ signatures (b) are presented, in addition to keeling plots, that is, the interrelation between $\delta^{13}C_{BC}$ vs 1/BC (c) and $\Delta^{14}C_{BC}$ vs 1/BC (d). Night-time only (01.00 to 06.00h, local time, GMT+3) samples collected during the high free-tropospheric aerosol loading events, June-July-August and December-January-February, were analyzed for carbon isotopes. The y-intercept in the keeling plot depicts the background BC source signature.

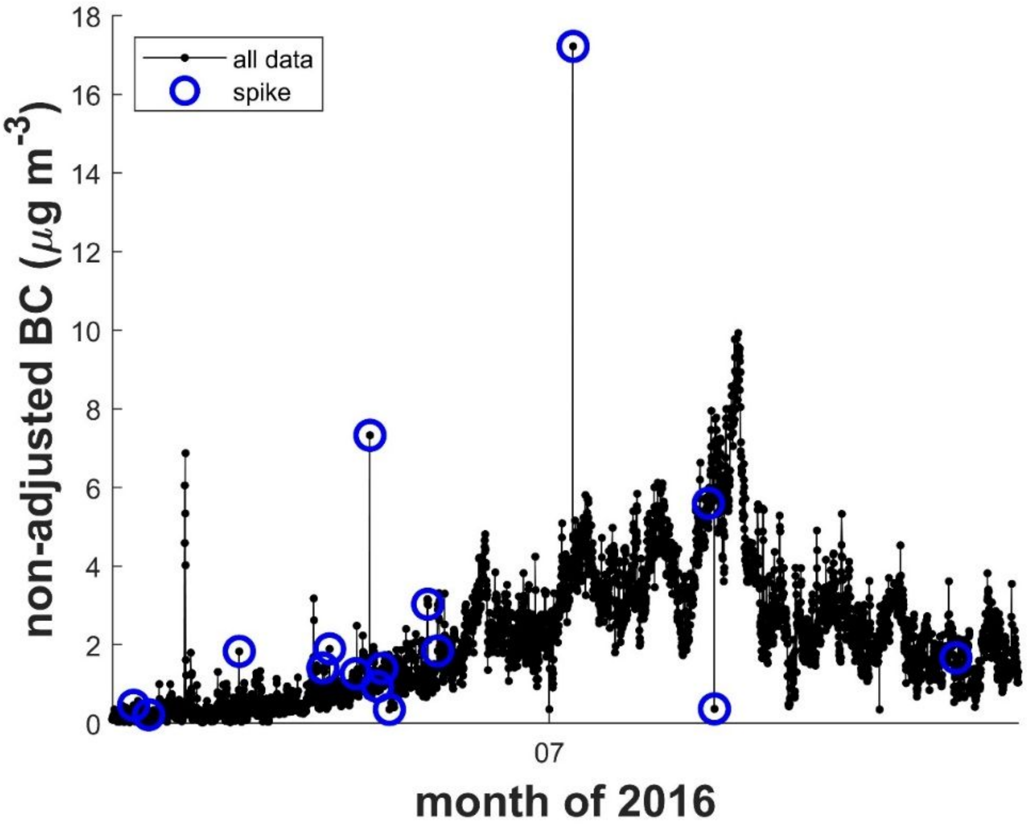

SI Figure S10: Illustration of spike-detection (blue circles) of the hourly resolution BC data from the aethalometer. The spikes in the hourly data potentially from short-term pollution events were removed by the following sliding window algorithm as described in SI Notes S1.

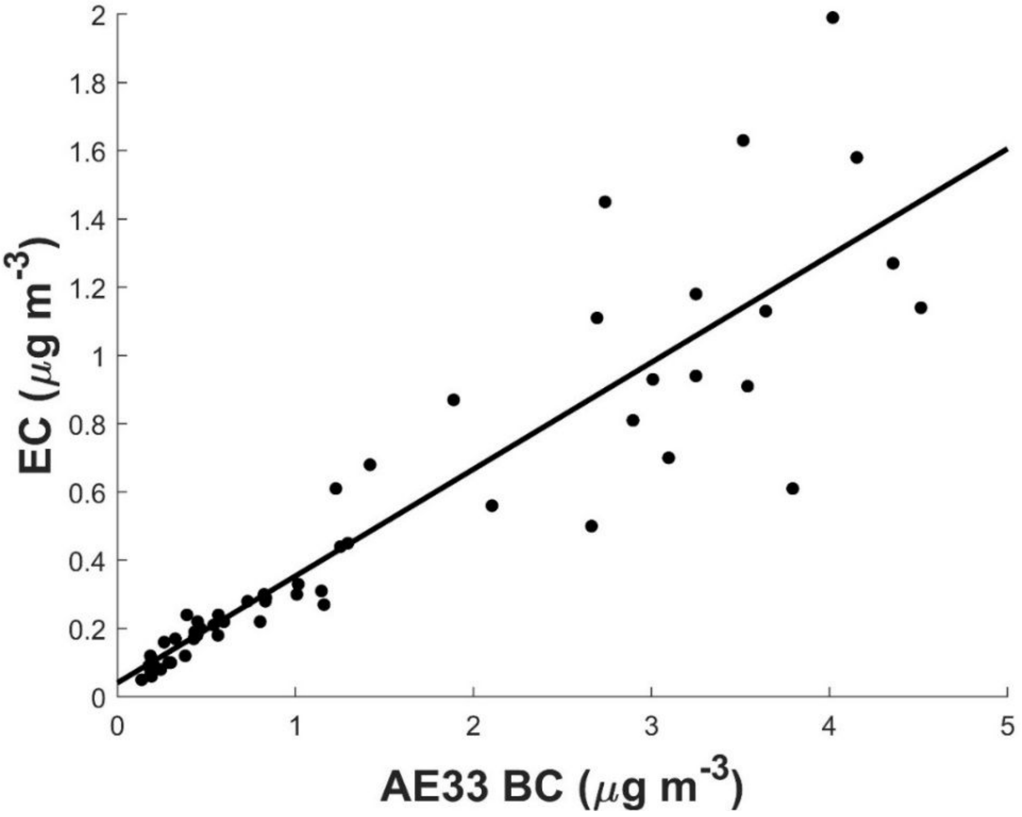

SI Figure S11: Correlation between eBC estimates from the AE33 aethalometer and sunset laboratory thermo-optical BC data. The aethalometer measurements were over-estimated by a factor of 3.2, possibly due to absorption enhancement of the aged plumes intercepted at Rwanda Climate Observatory.

References

(1) Drinovec, L.; Močnik, G.; Zotter, P.; Prévôt, A. S. H.; Ruckstuhl, C.; Coz, E.; Rupakheti, M.; Sciare, J.; Müller, T.; Wiedensohler, A.; Hansen, A. D. A. The "Dual-Spot" Aethalometer: An Improved Measurement of Aerosol Black Carbon with Real-Time Loading Compensation. *Atmos. Meas. Tech.* **2015**, *8* (5), 1965–1979. https://doi.org/10.5194/amt-8-1965-2015.

(2) Weingartner, E.; Saathoff, H.; Schnaiter, M.; Streit, N.; Bitnar, B.; Baltensperger, U. Absorption of Light by Soot Particles: Determination of the Absorption Coefficient by Means of Aethalometers. *J. Aerosol Sci.* **2003**, *34* (10), 1445–1463. https://doi.org/10.1016/S0021-8502(03)00359-8.

(3) Schmid, O.; Artaxo, P.; Arnott, W. P.; Chand, D.; Gatti, L. V.; Frank, G. P.; Hoffer, A.; Schnaiter, M.; Andreae, M. O. Spectral Light Absorption by Ambient Aerosols Influenced by Biomass Burning in the Amazon Basin. I: Comparison and Field Calibration of Absorption Measurement Techniques. *Atmos. Chem. Phys.* **2006**, *6* (11), 3443–3462. https://doi.org/10.5194/acp-6-3443-2006.

(4) Bond, T. C.; Doherty, S. J.; Fahey, D. W.; Forster, P. M.; Berntsen, T.; Deangelo, B. J.; Flanner, M. G. et al. Bounding the Role of Black Carbon in the Climate System: A Scientific Assessment. *J. Geophys. Res. Atmos.* **2013**, *118* (11), 5380–5552. https://doi.org/10.1002/jgrd.50171.

(5) Laing, J. R.; Jaffe, D. A.; Sedlacek, A. J. Comparison of Filter-Based Absorption Measurements of Biomass Burning Aerosol and Background Aerosol at the Mt. Bachelor Observatory. *Aerosol Air Qual. Res.* **2020**, *20* (4), 663–678. https://doi.org/10.4209/aaqr.2019.06.0298.

(6) Blanchard, D. C.; Woodcock, A. H. The Production, Concentration, and Vertical Distribution of the Sea-Salt Aerosol. *Ann. New York Acad. Sci.* **1980**, No. 954, 330–347.

(7) Millero, F. J.; Feistel, R.; Wright, D. G.; McDougall, T. J. The Composition of Standard Seawater and the Definition of the Reference-Composition Salinity Scale. *Deep. Res. Part I Oceanogr. Res. Pap.* **2008**, *55* (1), 50–72. https://doi.org/10.1016/j.dsr.2007.10.001.
